# Iatrogenic Cushing’s Syndrome and Fatal Adrenal Crisis Following Systemic Corticosteroid Therapy: A Case Series of Five Patients

**DOI:** 10.7759/cureus.112250

**Published:** 2026-07-08

**Authors:** Neeta Kurudagi, Basavaraj Sajjan, Soumya Morabad, S M Goornavar, Raghavendra S Hegde

**Affiliations:** 1 Pharmacy Practice, Basaveshwar Veerashaiva Vidyavardhaka (BVV) Sangha's Hanagal Shri Kumareshwar College of Pharmacy, Bagalkot, IND; 2 General Medicine, Shri Nijalingappa Medical College and Hanagal Shri Kumareshwar Hospital and Research Centre, Bagalkot, IND

**Keywords:** adrenal insufficiency, clinical pharmacy, corticosteroid withdrawal, drug safety, exogenous hypercortisolism, iatrogenic cushing’s syndrome, pharmacovigilance

## Abstract

Corticosteroids are indispensable in the management of inflammatory, autoimmune, and respiratory disease, yet prolonged or unsupervised systemic use can produce iatrogenic Cushing’s syndrome, and abrupt withdrawal risks life-threatening adrenal crisis. We report five male patients, aged 12 to 65 years, who developed Cushingoid features following systemic corticosteroid exposure for type two diabetes-associated dermatosis, bronchial asthma, epileptic encephalopathy, rheumatoid arthritis, and osteoarthritis. All five presented with moon facies; additional findings included purple striae, cutaneous thinning, abdominal hyperpigmentation, and impaired wound healing. Where measured, serum cortisol was low or low-normal, consistent with hypothalamic-pituitary-adrenal (HPA) axis suppression by exogenous steroid and thereby confirming an iatrogenic rather than endogenous origin. Opportunistic fungal infection masked by corticosteroid-mediated immunosuppression was a recurring complication. In one patient, abrupt discontinuation of dexamethasone precipitated adrenal crisis, multi-organ failure, and death. The remaining four patients recovered fully following structured tapering or physiological corticosteroid replacement. This report illustrates the clinical spectrum of iatrogenic hypercortisolism, emphasises the diagnostic significance of a suppressed endogenous cortisol, and underscores the imperative of gradual steroid withdrawal. It highlights the essential role of clinical pharmacists in rational prescribing, early adverse-effect recognition, and safe steroid tapering within a multidisciplinary care framework.

## Introduction

Glucocorticoids rank among the most widely prescribed drugs in clinical medicine, valued for potent anti-inflammatory and immunosuppressive effects across a broad range of conditions, including asthma, autoimmune disease, dermatological disorders, and neurological illness [1]. Their therapeutic benefit, however, is inseparable from a dose- and duration-dependent burden of adverse effects, of which iatrogenic Cushing's syndrome is among the most clinically significant and readily recognisable [2].

Iatrogenic Cushing's syndrome is estimated to affect up to 2% of patients receiving long-term systemic corticosteroid therapy, with prevalence rising substantially with higher doses and longer durations of treatment [2,3]. Cushing's syndrome describes the clinical state arising from chronic glucocorticoid excess. It may originate endogenously from a pituitary adenoma, adrenal tumour, or ectopic adrenocorticotropic hormone (ACTH) secretion, or exogenously from administered corticosteroids [2,3]. The exogenous form is the most commonly encountered cause in routine clinical practice, yet it remains under-recognised because its manifestations overlap substantially with the metabolic comorbidities, including obesity, hypertension, and glucose intolerance, that frequently prompted corticosteroid therapy in the first place [3,4].

A biochemical distinction of considerable practical importance separates exogenous from endogenous disease. In endogenous Cushing's syndrome, serum cortisol is elevated; in iatrogenic disease, the administered steroid suppresses the hypothalamic-pituitary-adrenal (HPA) axis, rendering endogenous cortisol characteristically low or undetectable even when the patient presents with florid Cushingoid features [5]. In resource-limited settings where advanced hormonal assays are unavailable, a low or low-normal morning cortisol in a patient with documented corticosteroid exposure and characteristic clinical features is supportive of an exogenous origin, though comprehensive endocrine evaluation, including ACTH stimulation testing, remains the standard of care when resources permit [5]. The same HPA axis suppression renders abrupt withdrawal hazardous, because the atrophied adrenal cortex cannot mount a physiological stress response and acute adrenal insufficiency may follow [5,6]. Children are particularly vulnerable, with a rapid onset of Cushingoid features and superimposed risks of growth suppression and developmental impairment [6,7]. The risk of HPA axis suppression is dose- and duration-dependent; potency of the corticosteroid agent and route of administration are additional determinants [7]. Current international guidance recommends gradual, individualised tapering of corticosteroids following prolonged use, with the rate determined according to dose, duration, potency, and clinical response [7,8].

We present five patients in whom iatrogenic Cushing's syndrome developed during systemic corticosteroid therapy for distinct underlying conditions. One patient died in association with abrupt steroid cessation. The series is presented to illustrate the breadth of clinical presentation, to highlight the supportive diagnostic value of morning cortisol measurement in distinguishing exogenous from endogenous disease, and to emphasise the preventive roles of structured tapering and clinical pharmacy involvement.

## Case presentation

Five patients were evaluated in the department of general medicine over a five-month observation period (January to June 2025). In each case, the working diagnosis of iatrogenic Cushing’s syndrome was based on a documented history of systemic corticosteroid exposure combined with characteristic clinical features. Where available, a low or low-normal morning serum cortisol provided supportive biochemical evidence; however, ACTH measurement and dynamic adrenal function testing were not performed in any patient due to resource constraints, and the diagnosis should therefore be understood as clinically supported rather than biochemically confirmed by current gold-standard criteria. Written informed consent for publication of clinical details and photographic images was obtained from each patient or, in the case of the fatal outcome, from the next of kin. A consolidated summary of all clinical, biochemical, dosing, tapering, and outcome data is provided in Table 1.

**Table 1:** Comprehensive clinical, biochemical, dosing, tapering, and outcome summary of five patients with iatrogenic Cushing’s syndrome Serum cortisol reference range was approximately 5-25 µg/dL (morning sample). All cortisol values were obtained as morning samples using CLIA. A low or low-normal cortisol in a patient with documented corticosteroid exposure and Cushingoid features is consistent with and supportive of HPA axis suppression by exogenous steroid; definitive confirmation requires ACTH stimulation testing. ACTH measurement and dynamic adrenal function testing were not performed in any patient due to resource constraints. Approximate cumulative doses are estimates based on reported doses and durations and were not independently verified from prescription records. Formal endocrine follow-up, including repeat cortisol measurement or ACTH stimulation testing, was not performed for any surviving patient. M: male; DM: diabetes mellitus; mmHg: millimetres of mercury; IV: intravenous; RBS: random blood sugar; OD: once daily; BD: twice daily; CLIA: chemiluminescence immunoassay; HPA: hypothalamic-pituitary-adrenal; Na: sodium; K: potassium

Parameter	Patient 1	Patient 2	Patient 3	Patient 4	Patient 5
Age (years)/sex	32/M	60/M	12/M	65/M	50/M
Underlying condition	Type 2 DM; chronic dermatosis	Bronchial asthma	Epileptic encephalopathy	Rheumatoid arthritis	Osteoarthritis (left tibia)
Indication for corticosteroid	Widespread cutaneous eruption	Bronchial asthma control	Epileptic encephalopathy	Rheumatoid arthritis	Osteoarthritis pain
Corticosteroid and route	Dexamethasone (injectable)	Dexamethasone (oral)	Prednisolone (oral)	Methylprednisolone (oral)	Prednisolone (oral)
Dose and frequency	4 mg once daily	0.5 mg twice daily	10 mg twice daily x 2 months; then 20 mg once daily x 3 months	4 mg once daily	30 mg twice daily
Duration of exposure	~Two months	~Three years	~Five months	~Eight months	~Two years
Approximate cumulative dose	~240 mg	~1,095 mg	~3,000 mg	~960 mg	~43,800 mg
Blood pressure (mmHg)	128/82	150/90	110/70	120/70	110/70
Serum cortisol (µg/dL) and assay details	24.72 (morning sample; CLIA, ~12 h after last dexamethasone dose)	Not performed	7.97 (morning sample; CLIA)	1.39 (morning sample; CLIA)	Not performed
Key Cushingoid features	Moon facies, cutaneous thinning, erythema (hands and abdomen), poor wound healing	Moon facies, abdominal hyperpigmentation	Moon facies, purple striae (Figure 3)	Moon facies, hyperglycaemia (RBS 254 mg/dL)	Moon facies, respiratory crepitations
Concurrent complications	Erythroderma; tinea incognito; right lower-limb ulcer (Figure 1)	Tinea incognito, chronic liver disease	Pneumonia	Spermatocele, mild hydrocele	Bronchopneumonia
Electrolytes at deterioration	Na: 128 mEq/L, K: 5.8 mEq/L (at the time of crisis)	Not applicable	Not applicable	Not applicable	Not applicable
Steroid management at admission	Abruptly discontinued	Controlled stepwise withdrawal	Switched to IV hydrocortisone (physiological replacement)	Transitioned to oral hydrocortisone	Structured stepwise prednisolone taper
Tapering strategy and rationale	Not applicable (abrupt cessation preceded crisis)	Alternate-day prednisolone 5 mg to allow gradual HPA recovery while maintaining asthma control	IV hydrocortisone 80 mg twice daily (acute cover); tapered as condition improved	Oral hydrocortisone 5 mg (morning 1 tablet, midday 1 tablet, evening half tablet) given markedly suppressed cortisol	Prednisolone 10 mg twice daily x 5 days, then once daily x 5 days, then half-dose morning x 5 days; stepwise to allow gradual adrenal recovery
Duration of hospital stay	Not applicable (died during admission)	Six days	10 days	Five days	Seven days
Clinical follow-up after discharge	Not applicable	Not formally documented beyond discharge	Not formally documented beyond discharge	15-day outpatient review; clinically satisfactory recovery	Not formally documented beyond discharge
Endocrine follow-up and repeat biochemistry	Not applicable	Not performed	Not performed	Not performed	Not performed
Outcome	Died-haemodynamic collapse, multi-organ failure	Recovered fully	Recovered fully; discharged without corticosteroid	Recovered fully	Recovered fully

Patient one

A 32-year-old man with type two diabetes mellitus maintained on metformin presented with generalised weakness, vomiting, and pruritic skin lesions of one year’s duration. Two months earlier, he had received injectable dexamethasone 4 mg once daily for approximately two months (approximate cumulative dose 240 mg) for a widespread cutaneous eruption. Examination revealed moon facies, diffuse cutaneous thinning, erythema over the abdomen and hands, and a poorly healing ulcer over the right lower limb (Figure 1). Blood pressure was 128/82 mmHg. Morning serum cortisol was 24.72 µg/dL, measured by chemiluminescence immunoassay (CLIA) approximately 12 hours after the last administered dose of dexamethasone. As dexamethasone is not cross-reactive with standard cortisol immunoassays, the measured value reflected residual endogenous cortisol output rather than total glucocorticoid load. In a patient receiving prolonged pharmacological-dose dexamethasone, a cortisol value in the low-normal range is consistent with partial but significant HPA axis suppression, because endogenous output would be expected to be near-zero under sustained exogenous glucocorticoid exposure. He was diagnosed with iatrogenic Cushing’s syndrome complicated by erythroderma secondary to tinea incognito and a healing right lower-limb ulcer. Dexamethasone was abruptly discontinued on day one of admission. Within 24 to 48 hours of cessation, the patient developed refractory hypotension unresponsive to intravenous fluid resuscitation, hypoglycaemia, and progressive obtundation. Laboratory findings included hyponatraemia (serum sodium 128 mEq/L) and hyperkalaemia (serum potassium 5.8 mEq/L), consistent with acute mineralocorticoid deficiency. Blood cultures obtained at this time were negative, making sepsis an unlikely primary precipitant. The temporal relationship between abrupt dexamethasone cessation and haemodynamic collapse, together with the electrolyte disturbances, is consistent with and supportive of adrenal crisis as the principal mechanism of deterioration, though definitive causal attribution in a clinical case report is inherently limited. Despite supportive treatment including antihistamine, antifungal, and antimicrobial therapy, the patient progressed to multi-organ failure and died. No steroid replacement was administered following abrupt cessation, and no endocrine follow-up was applicable.

**Figure 1:**
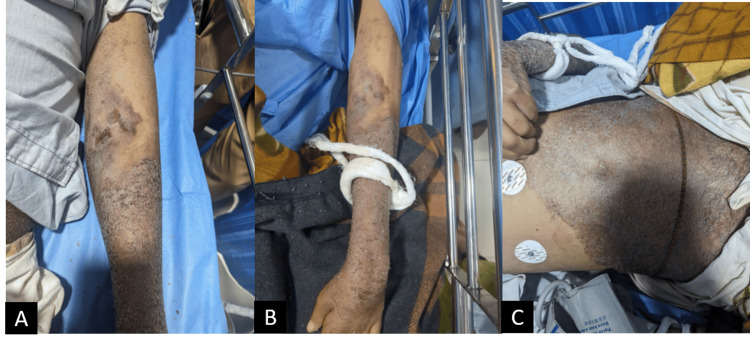
Patient one: cutaneous manifestations of iatrogenic Cushing's syndrome secondary to dexamethasone therapy (A) Right lower limb showing cutaneous thinning, hyperpigmentation, and poorly healing ulcerative skin lesions consistent with glucocorticoid-induced skin fragility and impaired wound healing. (B) Upper limb demonstrating cutaneous thinning and erythema with skin fragility; intravenous line is in situ, reflecting the patient's acute admission. (C) The truncal and abdominal region shows hyperpigmentation and skin thinning consistent with prolonged corticosteroid exposure. These findings, in a patient with a documented history of injectable dexamethasone use and characteristic moon facies, are consistent with and supportive of iatrogenic Cushing's syndrome in the appropriate clinical context. Written informed consent for publication of clinical details and images, including identifiable features, was obtained from each patient or, in the case of the fatal outcome in Patient 1, from the next of kin before submission. All patient data were de-identified to protect confidentiality.

Patient two

A 60-year-old man with bronchial asthma presented with abdominal distension and facial swelling of 25 days’ duration. He had been maintained on oral dexamethasone 0.5 mg twice daily for approximately three years (approximate cumulative dose 1,095 mg). Blood pressure was 150/90 mmHg. Examination showed moon facies and abdominal hyperpigmentation (Figure 2). Morning serum cortisol was not performed. He was diagnosed with acute exacerbation of asthma, iatrogenic Cushing’s syndrome, chronic liver disease, and tinea incognito. Dexamethasone was withdrawn in a controlled stepwise manner. The tapering strategy selected was alternate-day oral prednisolone 5 mg, chosen to allow gradual HPA axis recovery while maintaining sufficient corticosteroid cover to prevent acute asthma decompensation. He was managed with antimicrobial, hepatoprotective, diuretic, and bronchodilator therapy and was discharged after six days. Formal endocrine follow-up and repeat cortisol measurement were not performed beyond discharge.

**Figure 2:**
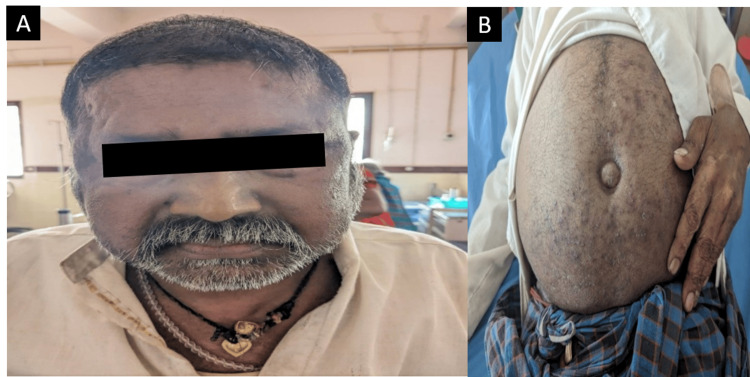
Patient two: moon facies and abdominal hyperpigmentation following prolonged dexamethasone therapy for bronchial asthma (A) Moon facies in a 60-year-old man with bronchial asthma maintained on oral dexamethasone for approximately three years, showing characteristic rounded facial contour and facial fullness consistent with glucocorticoid-induced fat redistribution. Eyes are de-identified to protect patient privacy. (B) Abdominal hyperpigmentation observed in the same patient, representing a less commonly reported cutaneous manifestation consistent with exogenous hypercortisolism. The co-existence of moon facies and abdominal hyperpigmentation in the setting of prolonged dexamethasone use is supportive of iatrogenic Cushing's syndrome. Written informed consent for publication of clinical details and images, including identifiable features, was obtained from each patient. All patient data were de-identified to protect confidentiality.

Patient three

A 12-year-old boy with epileptic encephalopathy presented with fever and cough of one day’s duration and facial swelling of 20 days. He had been maintained on oral prednisolone initiated at 10 mg twice daily for two months and subsequently increased to 20 mg once daily for three further months (total duration: approximately five months; approximate cumulative dose: 3,000 mg). Blood pressure was 110/70 mmHg. Examination demonstrated moon facies (Figure 3) and purple striae over the abdomen and limbs (Figure 3). Morning serum cortisol was 7.97 µg/dL, measured by CLIA. This value is low-normal and, in a symptomatic Cushingoid child with documented prednisolone exposure, is consistent with and supportive of HPA axis suppression; it makes endogenous hypercortisolism unlikely but cannot exclude it without ACTH stimulation testing, which was not performed. He was diagnosed with iatrogenic Cushing’s syndrome and concurrent pneumonia. The tapering strategy selected was intravenous hydrocortisone at physiological replacement dose, chosen to provide adequate glucocorticoid cover during acute illness while safely withdrawing the pharmacological prednisolone dose and allowing gradual HPA axis recovery. He was managed with antimicrobials and antiepileptic therapy and was discharged after 10 days without corticosteroids in the prescription. He recovered fully. Formal endocrine follow-up and repeat cortisol measurement were not performed beyond discharge.

**Figure 3:**
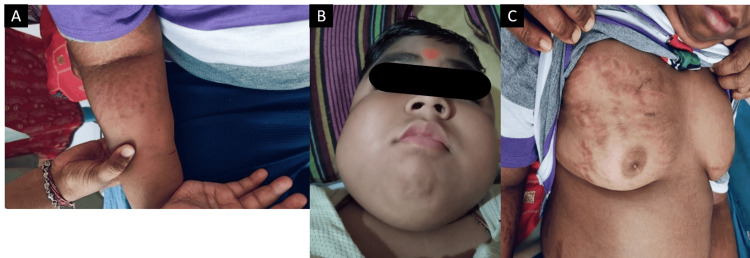
Patient three: purple striae, moon facies, and truncal skin changes in a paediatric patient with iatrogenic Cushing's syndrome secondary to prednisolone therapy (A) Purple striae over the forearm and hand in a 12-year-old boy maintained on oral prednisolone for epileptic encephalopathy, consistent with glucocorticoid-induced dermal atrophy and connective tissue catabolism. (B) Moon facies demonstrates a characteristic rounded facial contour and facial fullness consistent with cortisol-mediated fat redistribution. Face is de-identified to protect the identity of the minor patient. (C) Purple striae over the truncal and abdominal region, a well-recognised cutaneous manifestation consistent with hypercortisolism resulting from collagen breakdown induced by sustained glucocorticoid excess. The early development of these features in a paediatric patient is consistent with the heightened sensitivity of children to exogenous corticosteroids. Written informed consent for publication of clinical details and images, including identifiable features, was obtained from each patient or their parents/guardian. All patient data were de-identified to protect confidentiality.

Patient four

A 65-year-old man with rheumatoid arthritis presented with cough, breathlessness, facial swelling, and scrotal discomfort. He had been maintained on oral methylprednisolone 4 mg once daily for eight months (approximate cumulative dose: 960 mg). Blood pressure was 120/70 mmHg, and random blood glucose (RBS) was elevated at 254 mg/dL. Examination showed moon facies (Figure 4). Morning serum cortisol was 1.39 µg/dL, measured by CLIA. This markedly low value is consistent with and supportive of significant HPA axis suppression by exogenous corticosteroid; formal confirmation with ACTH stimulation testing was not performed due to resource constraints. He was diagnosed with iatrogenic Cushing’s syndrome with an incidental spermatocele and mild hydrocele. The tapering strategy selected was transition to oral hydrocortisone replacement at a physiological schedule (5 mg: one tablet morning, one tablet midday, half tablet evening), chosen given the markedly suppressed cortisol, indicating significant HPA axis compromise. He was managed with antimicrobial, analgesic, and insulin therapy and was discharged after five days. A 15-day outpatient review was conducted, during which clinical recovery was satisfactory. Formal endocrine follow-up with repeat cortisol measurement or ACTH stimulation testing was not performed.

**Figure 4:**
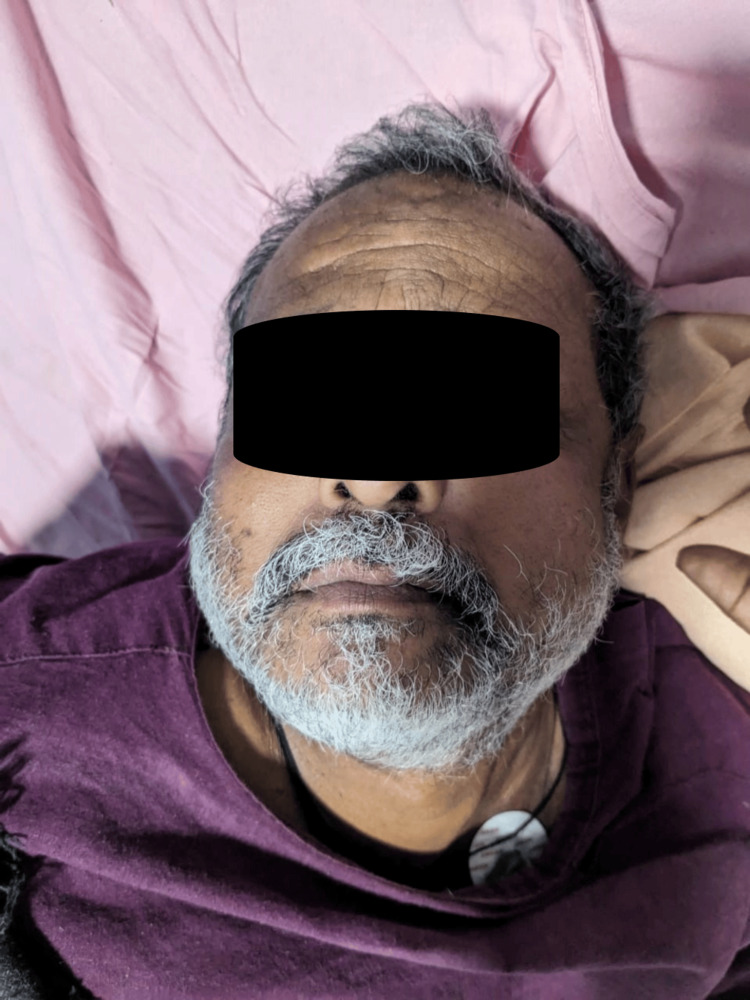
Patient four: moon facies in a patient with rheumatoid arthritis following eight months of methylprednisolone therapy Moon facies in a 65-year-old man maintained on oral methylprednisolone for rheumatoid arthritis for approximately eight months, demonstrating a characteristic rounded facial contour and periorbital fullness consistent with glucocorticoid-induced fat redistribution. Serum cortisol in this patient was 1.39 µg/dL (morning sample; chemiluminescence immunoassay), a markedly low value consistent with and supportive of significant hypothalamic-pituitary-adrenal axis suppression by exogenous corticosteroid; formal confirmation with ACTH stimulation testing was not performed. Face is de-identified to protect patient privacy. Written informed consent for publication of clinical details and images, including identifiable features, was obtained from each patient. All patient data were de-identified to protect confidentiality.

Patient five

A 50-year-old man with osteoarthritis of the left tibia presented with cough and breathlessness of 10 days and fever of seven days’ duration. He had been maintained on oral prednisolone 30 mg twice daily for approximately two years (approximate cumulative dose: 43,800 mg). Blood pressure was 110/70 mmHg, and respiratory examination revealed bilateral crepitations. Examination showed moon facies. Morning serum cortisol was not performed. He was diagnosed with iatrogenic Cushing’s syndrome with concurrent bronchopneumonia. The tapering strategy selected was a structured stepwise prednisolone reduction (10 mg twice daily for five days, then once daily for five days, then half the dose in the morning for five days), chosen to allow gradual adrenal recovery following two years of high-dose exposure while treating the acute respiratory illness. Intravenous hydrocortisone cover was provided during the acute illness. He was managed with antimicrobial and diuretic therapy and was discharged after seven days on the tapering regimen. He recovered fully. Formal endocrine follow-up and repeat cortisol measurement were not performed beyond discharge. No clinical images are presented for this case.

## Discussion

This series illustrates the clinical range of iatrogenic Cushing’s syndrome arising from systemic corticosteroid therapy across five distinct underlying diseases and five different corticosteroid regimens. Moon facies was a universal finding, with purple striae, cutaneous thinning, abdominal hyperpigmentation, and impaired wound healing observed variably across patients. These features reflect the catabolic and redistributive effects of sustained glucocorticoid excess on skin, connective tissue, and adipose compartments [2,8]. Metabolic disturbance accompanied the cutaneous picture: hypertension was documented in two patients and symptomatic hyperglycaemia in one, consistent with the established effects of cortisol on vascular tone, insulin sensitivity, and glucose metabolism [2].

The biochemical pattern in the three patients in whom cortisol was measured warrants careful interpretation. In patient four, the value of 1.39 µg/dL was markedly low; in patient three, 7.97 µg/dL was at the lower boundary of the reference range. Patient one’s value of 24.72 µg/dL, while nominally within the conventional reference range, required specific contextualisation: dexamethasone is not cross-reactive with standard CLIA cortisol assays, meaning the measured value reflected the residual endogenous output rather than total glucocorticoid load. In a patient receiving prolonged pharmacological-dose dexamethasone, a low-normal endogenous cortisol is consistent with partial but significant HPA axis suppression. Across all three cases, the important interpretive principle is that in exogenous Cushing’s syndrome, endogenous cortisol is suppressed by negative feedback on the HPA axis; a low or low-normal morning cortisol in this clinical context is therefore consistent with and supportive of exogenous disease [5]. This contrasts with endogenous Cushing’s syndrome, in which cortisol is elevated. We acknowledge that cortisol was measured in only three of five patients, that all values were single morning samples, and that ACTH measurement and dynamic adrenal function testing were not performed in any patient. Conclusions regarding biochemical patterns are therefore appropriately limited in their certainty and should be interpreted alongside the full clinical picture and current guideline recommendations [5].

The clinical course of patient one is the most instructive observation in this series. Following an abrupt discontinuation of dexamethasone 4 mg once daily after approximately two months of use, the patient developed haemodynamic collapse within 24 to 48 hours, with hyponatraemia (sodium 128 mEq/L), hyperkalaemia (potassium 5.8 mEq/L), hypoglycaemia, and refractory hypotension unresponsive to intravenous fluid resuscitation. Blood cultures were negative. The temporal sequence of events and the electrolyte disturbances were consistent with and supportive of adrenal crisis as the principal mechanism, though we acknowledge that definitive causal attribution is inherently limited in a descriptive case report and that other contributors to multi-organ failure cannot be entirely excluded. This observation is consistent with the established principle that abrupt cessation of prolonged corticosteroid therapy carries a risk of acute adrenal insufficiency and that gradual tapering is essential [5]. The four surviving patients, each of whom received either structured tapering or physiological hydrocortisone replacement, recovered without complication, an observation that further supports the importance of planned steroid withdrawal in this clinical context.

Opportunistic infection was identified in two of the five patients, both of whom harboured tinea incognito, whose characteristic inflammatory signs had been attenuated by corticosteroid-mediated immunosuppression, delaying recognition and antifungal treatment [9]. This observation illustrates a recognised hazard of chronic steroid therapy: the anti-inflammatory action of glucocorticoids can suppress the cardinal signs of infection, creating a misleading impression of dermatological stability. A low threshold for fungal investigation is warranted in any patient on prolonged systemic corticosteroids who presents with atypical skin lesions [9,10].

The paediatric case warrants specific comment. Children develop Cushingoid features more rapidly than adults and are at additional risk of growth suppression and neurodevelopmental consequences from prolonged pharmacological corticosteroid exposure [6,7]. The early appearance of moon facies and striae in a 12-year-old receiving prednisolone for epileptic encephalopathy underscores the importance of particular vigilance and the use of the lowest effective corticosteroid dose for the shortest necessary duration in this age group.

The observations across the five patients suggest that several adverse outcomes may have been preventable through rational prescribing at the lowest effective dose and shortest necessary duration, periodic clinical monitoring for Cushingoid features, structured tapering plans aligned with current guideline recommendations, and patient and caregiver education regarding the risks of self-discontinuation. These observations are descriptive and cannot establish causality or preventability with certainty, given the small sample size and the retrospective nature of the case series. Clinical pharmacists are well-positioned to support each of these preventive elements, contributing to dose optimisation, adverse-effect monitoring, and tapering plan implementation within the multidisciplinary team [1,7,8]. The potential contribution of clinical pharmacists is highlighted here as a recommendation informed by the clinical observations of this series rather than as a directly measured outcome.

Limitations

The diagnosis of iatrogenic Cushing’s syndrome in all five patients was clinically supported rather than biochemically confirmed by current gold-standard criteria, as ACTH measurement and dynamic adrenal function testing were not performed due to resource constraints. Morning serum cortisol was available in only three of five patients and was obtained as a single time-point measurement without dynamic testing. Exact cumulative corticosteroid doses are approximate estimates based on reported doses and durations and were not independently verified from prescription records. Formal endocrine follow-up, including repeat cortisol measurement or ACTH stimulation testing to assess HPA axis recovery, was not performed for any surviving patient; clinical follow-up beyond discharge was documented only for patient four at 15 days. Weight and body mass index (BMI) data at corticosteroid initiation and at presentation were not consistently documented and could not be reported. The series describes five patients at a single centre over five months and is intended to be illustrative and hypothesis-generating rather than definitive; no incidence or causality inferences are appropriate, and all findings should be interpreted in the context of current clinical guidelines and comprehensive endocrine evaluation when available [5,11].

## Conclusions

In this series of five patients, iatrogenic Cushing’s syndrome arising from prolonged systemic corticosteroid therapy presented across a range of underlying conditions and with a variety of clinical manifestations, of which moon facies was universally observed. In the three patients in whom morning cortisol was measured, low or low-normal values were consistent with and supportive of HPA axis suppression by exogenous steroid; these findings, interpreted alongside documented corticosteroid exposure and characteristic clinical features, supported the working diagnosis of exogenous hypercortisolism in each case. Comprehensive endocrine evaluation, including ACTH stimulation testing, remains the standard of care when resources permit and should be pursued where clinically feasible. The clinical course of patient one, in whom abrupt dexamethasone cessation was temporally associated with haemodynamic collapse and death, is consistent with the well-established risk of adrenal crisis following abrupt withdrawal of prolonged corticosteroid therapy and underscores the importance of planned, gradual tapering in accordance with current guidelines. The four surviving patients, each managed with structured tapering or physiological replacement, recovered fully, an observation that further supports guideline-recommended withdrawal strategies. Vigilant clinical monitoring, individualised tapering plans, judicious prescribing at the lowest effective dose, and the active involvement of clinical pharmacists in adverse-effect surveillance are recommended as essential components of safe corticosteroid management, based on the observations of this series and current evidence-based guidance.
